# Growth of *Mycobacterium tuberculosis* in vivo segregates with host macrophage metabolism and ontogeny

**DOI:** 10.1084/jem.20172020

**Published:** 2018-04-02

**Authors:** Lu Huang, Evgeniya V. Nazarova, Shumin Tan, Yancheng Liu, David G. Russell

**Affiliations:** 1 Department of Microbiology and Immunology, College of Veterinary Medicine, Cornell University, Ithaca, NY; 2 Department of Molecular Biology and Microbiology, Tufts University School of Medicine, Boston, MA

## Abstract

To understand how infection by *Mycobacterium tuberculosis* (Mtb) is modulated by host cell phenotype, we characterized those host phagocytes that controlled or supported bacterial growth during early infection, focusing on the ontologically distinct alveolar macrophage (AM) and interstitial macrophage (IM) lineages. Using fluorescent Mtb reporter strains, we found that bacilli in AM exhibited lower stress and higher bacterial replication than those in IM. Interestingly, depletion of AM reduced bacterial burden, whereas depletion of IM increased bacterial burden. Transcriptomic analysis revealed that IMs were glycolytically active, whereas AMs were committed to fatty acid oxidation. Intoxication of infected mice with the glycolytic inhibitor, 2-deoxyglucose, decreased the number of IMs yet increased the bacterial burden in the lung. Furthermore, in in vitro macrophage infections, 2-deoxyglucose treatment increased bacterial growth, whereas the fatty acid oxidation inhibitor etomoxir constrained bacterial growth. We hypothesize that different macrophage lineages respond divergently to Mtb infection, with IMs exhibiting nutritional restriction and controlling bacterial growth and AMs representing a more nutritionally permissive environment.

## Abstract (precis)

This study by Huang et al. demonstrates that lung macrophages of differing ontogeny respond divergently to *Mycobacterium tuberculosis* infection in vivo. Alveolar macrophages and interstitial macrophages adopt different metabolic states that promote or control *M. tuberculosis* growth, respectively.

## Graphical Abstract

**Figure:**
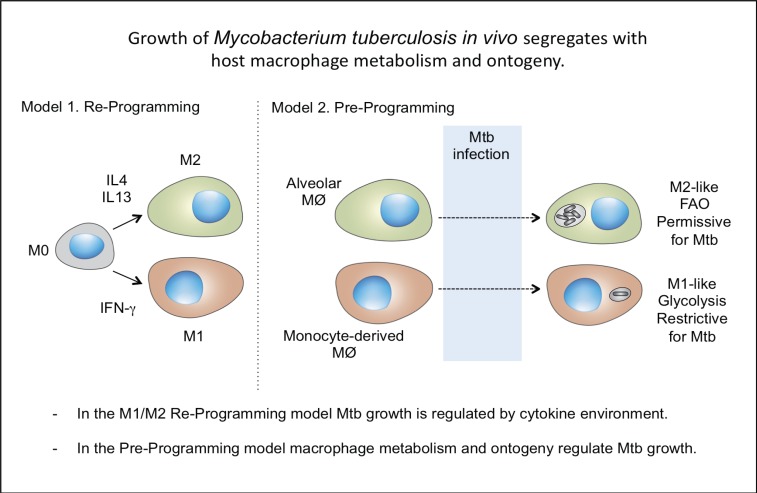

## Introduction

Under homeostatic conditions, macrophages derived from different ontogenies coexist in many tissues. In these situations, it is unclear the extent to which macrophage origin versus its tissue location and immune environment determines phagocyte phenotype and function. Some studies suggest that macrophages of different origins can exhibit both redundant and distinct functions in the same tissue in nondisease settings (Epelman et al., 2014; Gibbings et al., 2015; van de Laar et al., 2016). These data indicate that both tissue niche and cell origin contribute significantly to macrophage function at steady state. However, the relative impact of tissue environment versus cellular ontogeny on the progression of infectious disease remains poorly understood.

*Mycobacterium tuberculosis* (Mtb) resides in lung phagocytes and can persist in its host for decades. Macrophages are the most abundant host cells at sites of infection and have been implicated in both disease control and progression (Flynn et al., 2011). Two major macrophage populations have been described in the lung: alveolar macrophages (AMs) and interstitial macrophages (IMs). AMs are fetal liver derived during embryogenesis and are capable of self-renewal at steady state (Guilliams et al., 2013). In contrast to AMs, IMs have been less well characterized and are generally thought to arise from monocytes at steady state (Tan and Krasnow, 2016; Gibbings et al., 2017). Several studies using phenotypic markers to define IMs through expression of the integrins CD11c and CD11b have indicated these cells are recruited during Mtb infection (Srivastava et al., 2014).

Recent data from nonhuman primates suggest that infection outcome may correlate with the relative proportion of alternatively (M2) versus classically (M1) activated macrophages (Marino et al., 2015). This is consistent with previous research demonstrating that manipulation of lung macrophage populations can have a profound influence, both positively and negatively, on bacterial burden (Leemans et al., 2001; Antonelli et al., 2010). These earlier studies imply that, in addition to immune control, the permissiveness of lung macrophages to bacterial growth may also contribute to disease progression. We therefore contend that determining the relative fitness and replication status of Mtb within the different host macrophage populations at sites of infection is critical to identifying those host cells that would best favor progression to active disease. Furthermore, data strongly suggest that the heterogeneity of lung macrophages, and their distinct capabilities for controlling bacterial growth in Mtb infection, are critical in determining whether lung macrophages of different origins experiencing comparable inflammatory conditions exhibit divergent responses to the pathogen.

In this study, we exploit fluorescent Mtb fitness reporter strains to functionally define the relationship between the bacterium and the different host macrophage lineages present in the infected mouse lung. The fluorescent Mtb reporter strains, which we have characterized previously (Tan et al., 2013; Sukumar et al., 2014), demonstrate that bacilli residing in AMs exhibit lower stress and higher replication rates than those in IMs. The significance of this difference for the magnitude of the bacterial load was demonstrated experimentally through the selective depletion of AMs and IMs. Finally, AMs and IMs revealed dramatically different transcriptional profiles and acquire metabolically distinct states. IMs are highly glycolytically active, whereas AMs are up-regulated for fatty acid uptake and β-oxidation. Intracellular Mtb is known to access and use fatty acids and cholesterol from the host cell (Lee et al., 2013; Podinovskaia et al., 2013; VanderVen et al., 2015; Nazarova et al., 2017), suggesting that those bacteria in AMs would be at a metabolic advantage. Nutritional immunity is a concept based on immune modulation of iron sequestration (Kochan, 1973); however, the current data indicate a similar linkage between host cell phenotype and nutrient availability. More importantly, these data demonstrate the critical role that macrophage ontogeny plays in the localized response to early tuberculosis infection.

## Results

### Mtb infection induces accumulation of lung IMs

We chose to focus on a 2-wk time point because (1) it is a period of rapid bacterial expansion, (2) the bacteria are relatively equally distributed between the AM and IM populations, and (3) the early innate immune response is well established (North and Jung, 2004). The study is not intended to model disease progression but rather to explore the significance of host phagocyte populations at the site of infection.

**Figure 1.**
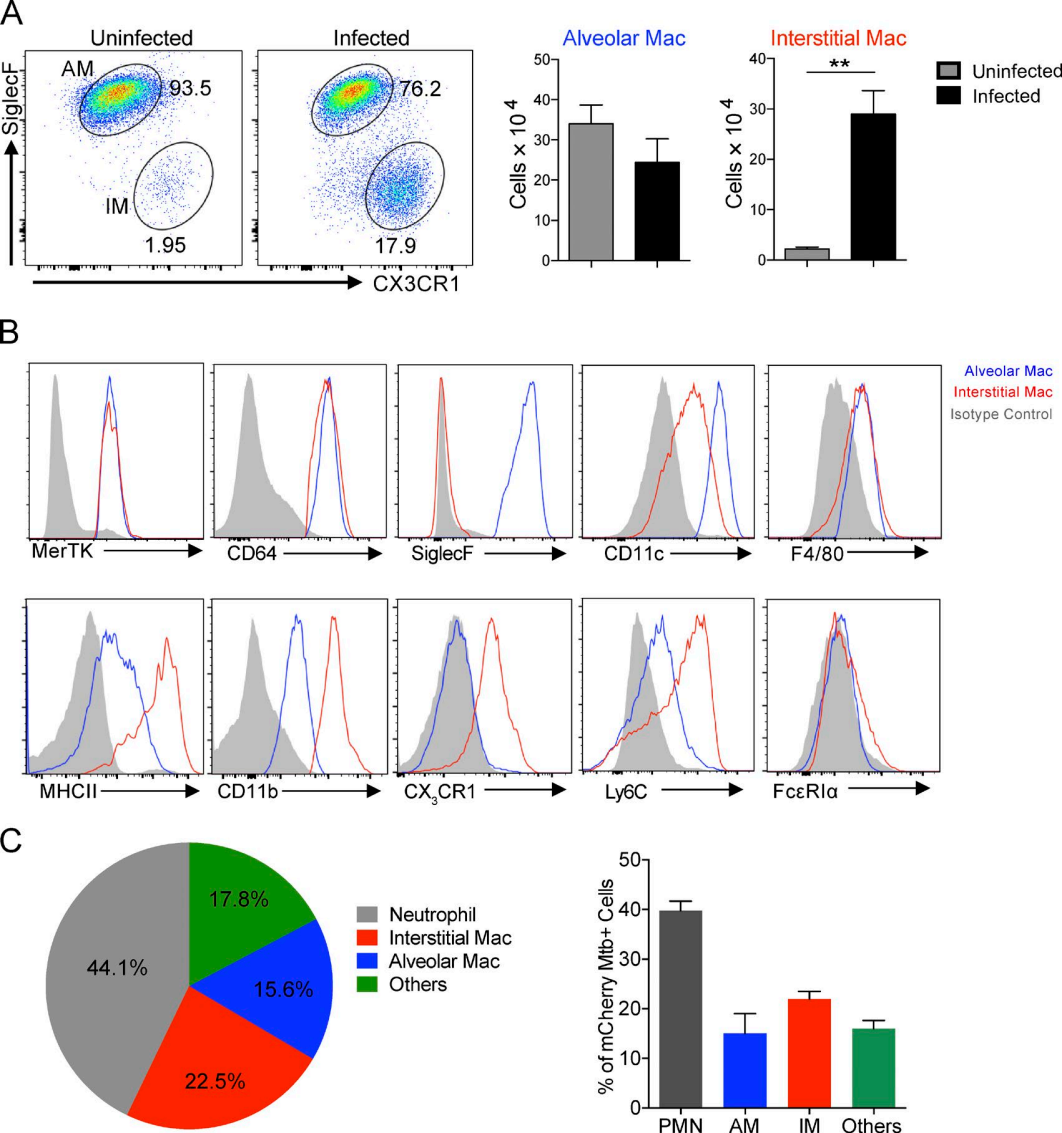
**Mtb infection induces accumulation of interstitial macrophages.** To characterize lung macrophage populations and determine the relative distribution of Mtb within the phagocyte subsets of the murine lung, tissue was harvested from WT mice 2 wk after infection by intranasal challenge with 10^3^ Mtb. The phagocyte subsets were analyzed by flow cytometry to determine their identity and infection status. **(A)** Flow cytometry analysis of lung macrophage (Mac) numbers. Mean ± SD are shown in the column bar graph. *n* = 5. P-value was calculated using Student’s *t* test. **, P < 0.01. **(B)** Flow cytometry analysis of lung macrophage phenotype. Blue, AMs; red, IMs; gray, isotype control. **(C)** Flow cytometry analysis of distribution of Mtb in lung phagocytes. mCherry Mtb were used in the experiment. Mean from at least three mice are shown in the pie chart. Mean ± SD are shown in the column bar graph. The experiments were repeated three times.

We developed an inclusive flow cytometric gating protocol based on previous analysis of the different types of lung phagocytes present during the early stages of Mtb infection (Fig. S1). Coexpression of MerTK and CD64 specifically identifies all macrophages in various organs, including the lung (Gautier et al., 2012; Gibbings et al., 2017). The expression of Siglec F and CD11c enabled us to further identify two major types of macrophages in the lungs of mice during Mtb infection, namely AMs and IMs. Strikingly, whereas the number of AMs 2 wk postinfection was marginally reduced in the infected versus uninfected lung, we observed a large accumulation of IM in the infected lung (Fig. 1 A). 2 wk after Mtb infection, both types of macrophages expressed low levels of F4/80 but high levels of the tissue macrophage markers CD64 and MerTK (Fig. 1 B). However, AMs were Siglec F^+^ and CD11c^high^, whereas IMs were Ly6C^high^, CX_3_CR1^+^, and CD11b^high^, suggesting that they were monocyte derived. Both AMs and IMs did not express FcεRIα (Fig. 1 B), a marker of monocyte-derived dendritic cells (Guilliams et al., 2014). We next assessed the distribution of Mtb in the lung phagocyte populations. 2 wk after infection, the majority of Mtb bacteria were present in neutrophils. More than 20% and 15% of Mtb bacteria were harbored in IMs and AMs, respectively (Fig. 1 C).

### Lung IMs derive from recruited monocytes during Mtb infection

**Figure 2.**
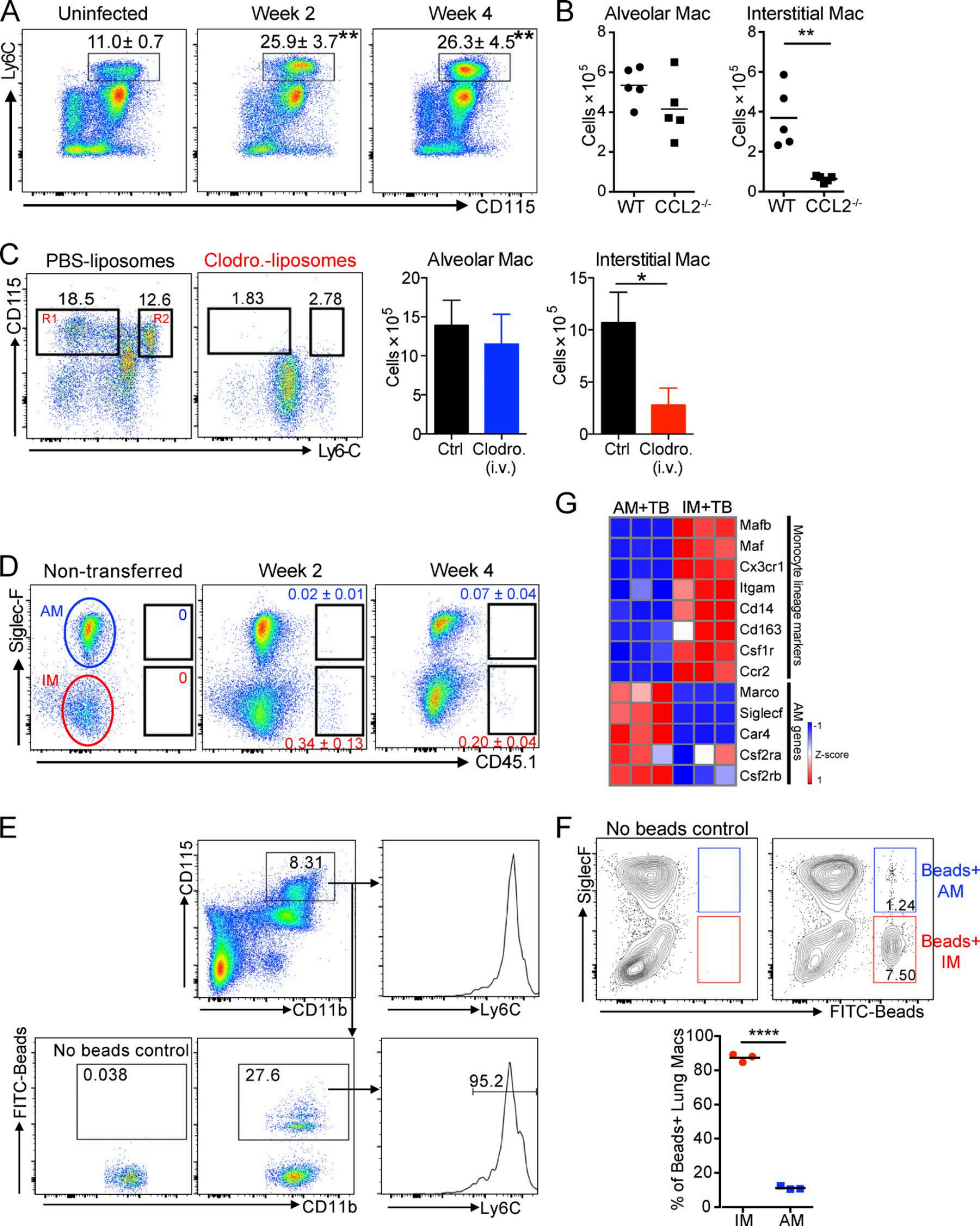
**Recruited monocytes give rise to lung interstitial macrophages in Mtb infection.** Accumulation of IMs in infected lung tissue is impaired in CCL2^−/−^ mice and in mice that have been treated with clodronate liposomes to deplete their circulating monocytes. Moreover, cell transfer experiments with congenic CD45.1 mice and selective labeling of blood monocytes with FITC-bead experiments demonstrate that IMs are derived from the circulating monocyte population. **(A)** Flow cytometry analysis of blood monocytes gated on CD45^+^CD11b^+^ cells. *n* = 3. The experiment was repeated two times. Mean ± SD are shown in the flow plots. Asterisks indicate statistical significance in comparison to uninfected mice. P-values were calculated using Student’s *t* test. **, P < 0.01. **(B)** Numbers of AMs and IMs in WT and CCL2^−/−^ mice. The experiment was repeated two times. *n* = 5. P-value was calculated using Student’s *t* test. **, P < 0.01. **(C)** Numbers of AMs and IMs in mice treated with clodronate liposomes intravenously. R1, Ly6C^low^ monocytes; R2, Ly6C^hi^ monocytes. PBS liposomes or clodronate liposomes were given to mice every 2 d. The experiment was repeated two times. *n* = 3–5. Error bars are standard deviation. P-value was calculated using Student’s *t* test. *, P < 0.05. **(D)** Flow cytometry analysis of lung macrophages obtained from WT mice. Mice were transferred with bone marrow monocytes isolated from CD45.1 mice at 10 and 24 d after infection. Lung cells were obtained at 2 and 4 wk after infection. The experiment was repeated three times. *n* = 4. **(E and F)** Mtb-infected mice were treated with clodronate-loaded liposome intravenously, followed by intravenous injection of FITC beads. **(E)** FITC-bead–labeled Ly6C^hi^ blood monocytes 24 h after bead injection. **(F)** Flow cytometry analysis of FITC-bead–labeled lung macrophage populations 72 h after bead injection (gated on live, Ly6G^−^MertK^+^CD64^+^ cells). *n* = 3. The experiment was repeated two times. P-value was calculated using Student’s *t* test. ****, P < 0.0001. (B and F) Horizontal bars indicate the mean. **(G)** Heat map showing relative expression of monocyte- and AM-lineage–associated genes.

We next aimed to investigate the origin of IMs and AMs in the Mtb-infected lung. It is accepted that Mtb infection leads to recruitment of blood monocytes that further differentiate into various pulmonary phagocytes, including macrophages (Sköld and Behar, 2008; Antonelli et al., 2010; Samstein et al., 2013; Srivastava et al., 2014). We first examined monocyte numbers in the blood of naive and infected mice. We found that Mtb-infected mice developed monocytosis, as evidenced by increased numbers of Ly6C^hi^CD11b^+^CD115^+^ cells in the blood at 2 and 4 wk after infection (Fig. 2 A). Furthermore, the accumulation of IMs, but not AMs, in the infected lung is impaired in the absence of CCL2 (Fig. 2 B), a chemokine that is responsible for mobilization/recruitment of monocytes to the blood and lung during infection (Peters et al., 2001; Hohl et al., 2009; Samstein et al., 2013). Depletion of blood monocytes through intravenous injection of clodronate-loaded liposomes (Claassen et al., 1990) abolished the accumulation of IMs without impacting the AM population in the Mtb-infected lung, suggesting that blood monocytes were the primary source of IMs (Fig. 2 C). In a complementary approach, we transferred monocytes isolated from CD45.1 mice to infected WT recipient mice intravenously on days 10 and day 24 after infection. 2 and 4 wk after infection, CD45.1^+^ IMs were detected, indicating that monocytes give rise to IMs, but not AMs, during the infection (Fig. 2 D). We further assessed the contribution of blood monocytes to lung macrophage populations during Mtb infection by selectively labeling Ly6C^hi^ monocytes in the blood with fluorescently labeled beads (Tacke et al., 2006). FITC beads were detected in 27% of Ly6C^hi^ monocytes in the blood upon its reconstitution after clodronate-loaded liposome depletion (Fig. 2 E). Nearly 90% of the bead-labeled monocytes differentiated into the IM population of the lung at 2 wk after infection (Fig. 2 F). Lastly, transcriptional profiling data demonstrated that the monocyte lineage markers such as CX_3_CR1, CCR2, CD14, and CD163 were highly expressed on IMs compared with AMs (Fig. 2 G). In contrast, unique cell surface molecules for identification of AM, including CSF2R, SiglecF, and MARCO, were absent in IMs (Fig. 2 G). Together, these data show that Mtb infection triggers the accumulation of IMs derived from blood monocytes that are distinct from the resident AM population.

### Mtb infection arrests cell cycle of lung macrophages but induces the local proliferation of uninfected bystander lung macrophages

**Figure 3.**
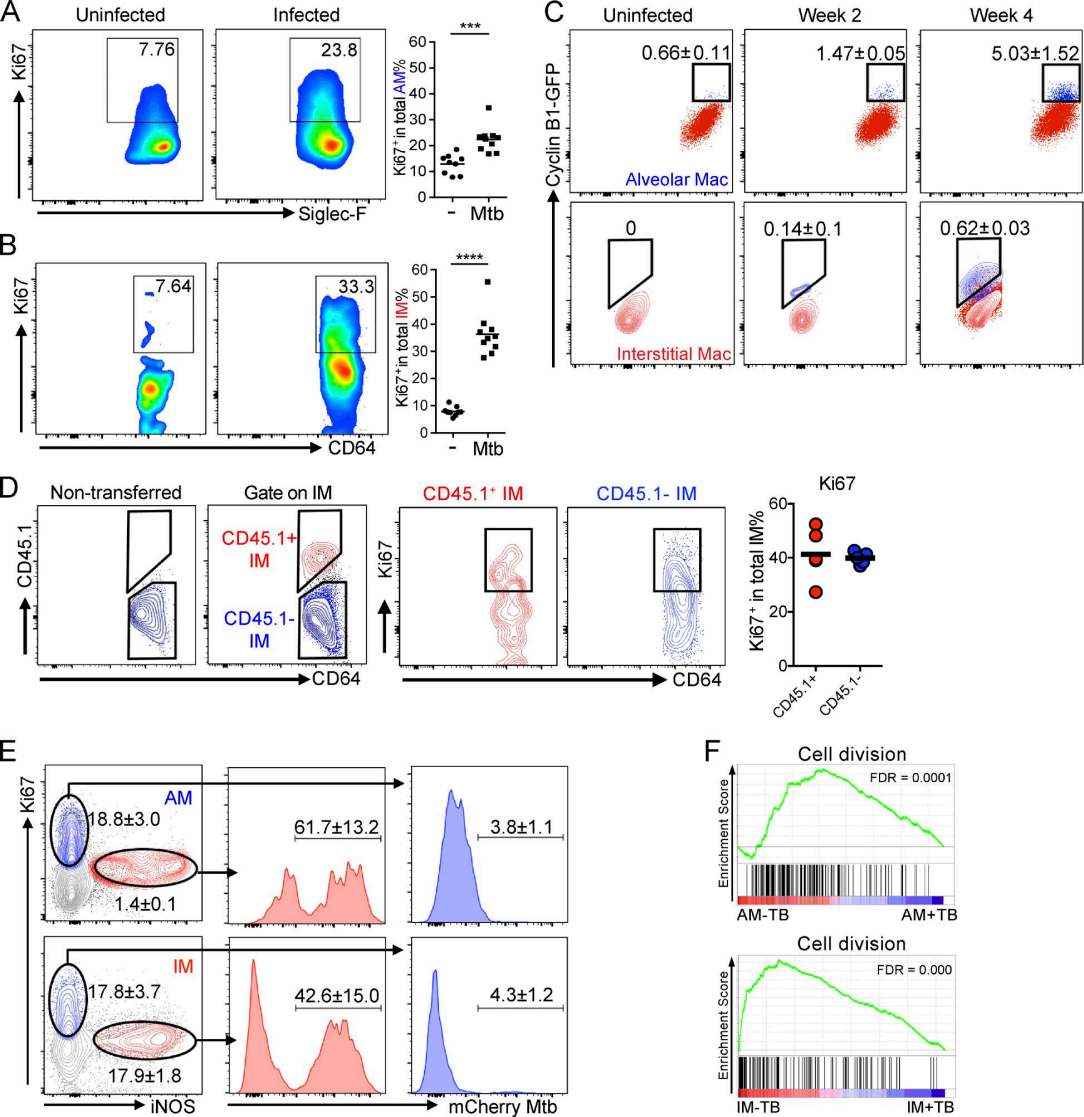
**Mtb induces local proliferation of lung macrophages.** Lung cells isolated at indicated time points were probed with an antibody against the nuclear replication marker Ki67. Both uninfected bystander AMs and IMs exhibited an increased expression in Ki67 2 wk after infection. **(A and B)** Flow cytometry analysis of lung macrophages. Percentages of Ki67^+^ cells in AM and IM populations gated as described in Fig. S1 were determined. *n* = 9. The experiment was repeated three times. P-values in A and B were calculated using Student’s *t* test. ***, P < 0.001; ****, P < 0.0001. **(C)** Flow cytometry analysis of lung macrophages in Cyclin B1-GFP mice 2 and 4 wk after infection. The experiment was repeated two times. Mean ± SD are shown in the flow plots. *n* = 3. **(D)** Flow cytometry analysis of lung macrophages obtained from WT mice. Mice were transferred with bone marrow monocytes isolated from CD45.1 mice 10 d after infection. Lung cells were obtained 2 wk after infection. *n* = 4. The experiment was repeated three times. (A, B, and D) Horizontal bars indicate the mean. **(E)** Flow cytometry analysis of iNOS and Ki67 expression in gated AMs and IMs 2 wk after infection. The experiment was repeated three times. Mean ± SD are shown in the flow plots. *n* = 3. **(F)** GSEA enrichment plots of AM-TB versus AM+TB and IM-TB versus IM+TB with false discovery rates (FDRs).

Published studies indicate that both AM and IM are capable of proliferation in situ at steady state (Gibbings et al., 2017). To determine whether local proliferation contributes to the accumulation of lung macrophages in Mtb infection, we analyzed Ki67 expression in both AMs and IMs. In uninfected mice, both AMs and lung resident IMs display a basal level of self-renewal capacity, as evidenced by Ki67 labeling (Fig. 3, A and B). In contrast, upon Mtb infection, replicating Ki67^+^ AMs and IMs increased dramatically 2 wk after infection (Fig. 3, A and B). This replication status was verified in an independent experiment using Cyclin B1-GFP transgenic mice to fluorescently “tag” replicating cells in vivo (Klochendler et al., 2012). Consistent with the Ki67 staining results, Mtb infection triggered increased numbers of GFP-positive AMs and IMs at both 2 and 4 wk after infection (Fig. 3 C). Notably, although inducible nitric oxide synthase (iNOS) activity was reduced in both AM and IM in Rag2^−/−^ mice, replication activity of AM and IM was comparable in Mtb-infected WT and Rag2^−/−^ mice, indicating that this process is independent of adaptive immunity and unlikely to be regulated by iNOS expression alone (Fig. S2, A and B). Moreover, CD45.1^+^ IM differentiated from adoptive transferred monocytes expressed levels of Ki67 comparable with recipient IMs, indicating that monocyte-derived IMs undergo active proliferation during Mtb infection (Fig. 3 D). These data demonstrate that, in addition to recruitment of blood monocytes, local macrophage proliferation also contributes to accumulation of macrophages in the lung during Mtb infection.

Recent data indicate that Mtb infection arrests replication of its host cell (Cumming et al., 2017). Consistent with this, we observed that nearly all Mtb-infected cells are Ki67 negative (Fig. 3 E), indicating that the infection is increasing proliferation of uninfected bystander macrophages. In addition, expression of iNOS is greater in IMs, and our analysis demonstrated that expression of Ki67 and iNOS was mutually exclusive in both AM and IM populations and that Mtb was found only in iNOS^+^, Ki67^−^ cells (Fig. 3 E). Gene set enrichment analysis (GSEA) further revealed that genes involved in cell division were enriched in uninfected bystander AMs and IMs compared with infected cells (Fig. 3 F), confirming the arrested cell cycle in Mtb-infected lung macrophages.

### Reporter Mtb strains reveal distinct bacterial fitness in lung phagocytes

**Figure 4.**
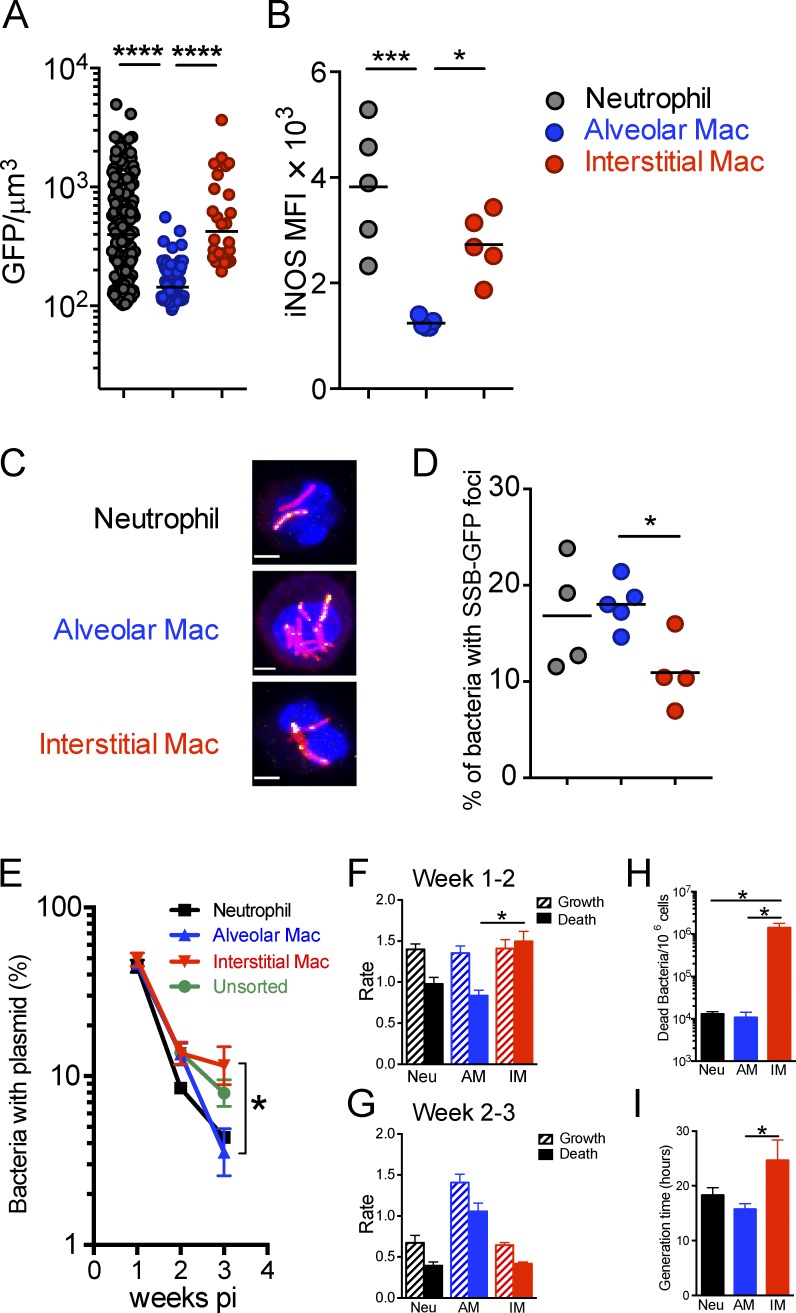
**Reporter Mtb strains reveal distinct fitness states of Mtb in lung phagocytes.** The fitness and replication status of Mtb in the different host phagocyte subsets was determined using fluorescent Mtb reporter strains and Mtb carrying a “clock” plasmid. The results indicate that AMs are markedly more permissive to Mtb and IMs. In addition, Mtb in neutrophils, although more stressed, appear to be replicating. Lungs were harvested from WT mice 2 wk after infection after intranasal infection with 10^3^ *hspX*’::GFP, *smyc’*::mCherry Mtb or SSB-GFP, *smyc’*::mCherry Mtb. Neutrophils, AMs, and IMs were FACS sorted from lung cell suspension. **(A)** Quantification of the GFP/µm^3^ signal from each bacterium measured from multiple 3D confocal images. Mice were infected with *hspX*’::GFP, *smyc’*::mCherry Mtb. *n* = 3. The experiments were repeated two times. Horizontal bars indicate the median. **(B)** Flow cytometry analysis of iNOS expression in lung phagocytes. *n* = 5. The experiments were repeated two times. **(C)** 3D confocal images of lung phagocytes sorted from SSB-GFP, *smyc’*::mCherry Mtb infected mice. Bars, 3 µm. **(D)** Percentage of Mtb displaying SSB-GFP foci for each cell type. Each point represents one experiment with four or five mice. The results were pooled from five experiments. P-values in A, B, and D were calculated using one-way ANOVA followed by Tukey’s post hoc tests. *, P < 0.05; ***, P < 0.001; ****, P < 0.0001. (B and D) Horizontal bars indicate the mean. **(E)** The percentage of bacteria containing the pBP10 plasmid in unsorted samples (green line) neutrophils (black line), AMs (blue line), and IMs (red line). Mean ± SEM are shown. The experiments were repeated three times. *n* = 3–5 mice. P-value was calculated using Student’s *t* test. *, P < 0.05. **(F and G)** Calculated growth (hash bar) and death rates (solid bar) during indicated intervals. Mean ± SD are shown. The experiments were repeated three times. *n* = 3–5 mice. P-value was calculated using Student’s *t* test. *, P < 0.05. **(H and I)** Calculated dead bacteria per 10^6^ cells of indicated cell type (H) and bacteria generation time (I) during 1–3 wk of infection. The experiments were repeated three times. *n* = 3–5 mice. P-values were calculated using Student’s *t* test. *, P < 0.05.

Given the emerging information of the ontogeny of the different phagocyte populations in the infected mouse lung, we sought to determine Mtb’s fitness and replication status in the different lung macrophage populations using the fluorescent Mtb reporter strains employed previously to probe the immune environment of the infected mouse (Sukumar et al., 2014). To assess the stress response of Mtb in different lung phagocytes, we infected mice with the *hspX*’::GFP, *smyc’*::mCherry reporter strain, which expresses GFP in response to nitric oxide in vivo. Infected neutrophils, AM and IM were sorted and the intensity of GFP signal quantified. We observed higher levels of *hspX*’::GFP fluorescence in Mtb present in neutrophils and IM, compared with those in AM (Fig. 4 A). As expected, the level of GFP expression in Mtb correlated with iNOS expression in the three types of phagocytes (Fig. 4 B). To determine if the different host phagocyte populations impact Mtb’s ability to replicate, we probed the replication status of Mtb in the different phagocytes by using the fluorescent replication reporter, SSB-GFP, which localizes to the chromosomal replisome during active DNA replication (Sukumar et al., 2014). 2 wk after infection, neutrophils, AMs and IMs were flow-sorted and SSB-GFP foci in Mtb quantified by confocal microscopy (Fig. 4 C). We observed significantly fewer SSB-GFP foci in Mtb within IMs in comparison to those bacilli in neutrophils and AMs (Fig. 4 D).

To provide independent validation of the SSB-GFP replication data we used Mtb-harboring the replication clock plasmid pBP10, which is lost from Mtb at a fixed, replication-dependent rate (Gill et al., 2009; Rohde et al., 2012). The rate of plasmid loss and CFU data allow us to calculate Mtb replication and death rates in the host cell subsets. During weeks 1–2, the loss of pBP10 plasmid in Mtb recovered from neutrophils, AMs, and IMs indicated rapid replication (Fig. 4 E). Correspondingly, the replication rate of Mtb was comparable among the three types of host cells during weeks 1–2 of infection (Fig. 4 F). In marked contrast, there was a striking increased death rate of Mtb in IMs compared with AMs (Fig. 4 F), indicating a greater capacity of these cells to control Mtb. In weeks 2–3, the proportion of Mtb with pBP10 plasmid was further reduced in AMs, whereas a greater number of Mtb harboring the pBP10 plasmid were recovered from IMs (Fig. 4 E), suggesting that IMs are also more capable of limiting Mtb replication during this time frame. Consistently, we found that bacterial replication rate was much slower in IMs versus AMs during this time period (Fig. 4 G). Extrapolation from these data using the mathematical model developed by Gill et al. (2009) indicates that the rate of bacterial death was significantly higher in the Mtb population within IMs in comparison to those bacteria in neutrophils and AMs over the first 3 wk of infection (Fig. 4 H). These data also indicate that the generation time for Mtb was markedly greater for those bacteria resident in IMs (Fig. 4 I).

The clock plasmid data report the cumulative history of the bacterial populations without providing information on host cells occupied before the time point of analysis. In contrast, the SSB-GFP and *hspX*’::GFP reporter strains provide a snapshot of host cell environment at time of analysis. Together, these datasets strongly suggest that AMs represent a more permissive cell type for replication and survival of Mtb. In contrast, IMs appear to restrict Mtb replication and be responsible for a greater number of bacterial deaths over the course of the infection period.

These experiments exploited bacterial replication and fitness reporters to define host cell phenotypes as permissive (AMs) or restrictive (IMs) and demonstrate that these functional phenotypes could be assigned specifically to the AM and IM populations.

### Depletion of macrophage subsets differentially influences bacterial burden

**Figure 5.**
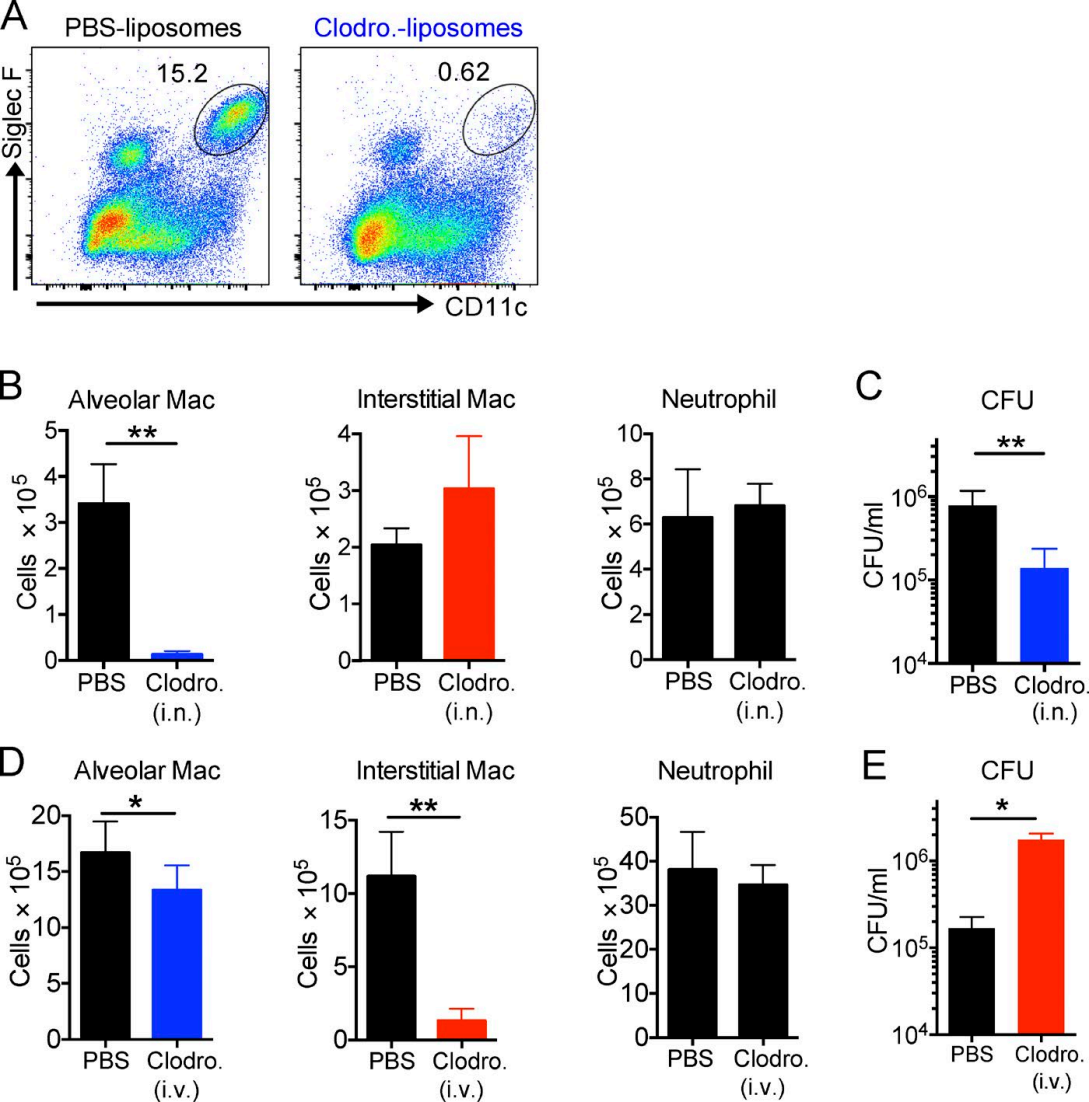
**Selective depletion of AMs and IMs results in a decrease or an increase in bacterial burden, respectively.** Treatment of mice by clodronate liposomes delivered either i.n. or i.v. to deplete the AMs or circulating monocytes, respectively, resulted in a reduction (AM-depleted) or an expansion (IM-depleted) in the bacterial burden, measured 2 wk after infection. **(A)** AM depletion upon intranasal treatment of clodronate liposomes. Liposomes were given to mice intranasally every day for the first week and every 2 d for another week. *n* = 5. The experiment was repeated two times. **(B)** Numbers of AMs, IMs, and neutrophils in mice treated with clodronate liposomes i.n. P-value was calculated using Student’s *t* test. **, P < 0.01. **(C)** Mtb bacterial burden in the lung of Mtb-infected mice treated with PBS liposomes or clodronate liposomes i.n. (depletion of AMs). P-value was calculated using Student’s *t* test. **, P < 0.01. **(D)** Numbers of neutrophils, AMs, and IMs in mice treated with clodronate liposomes i.v. *n* = 3–5. The experiment was repeated two times. P-values were calculated using Student’s *t* test. *, P < 0.05; **, P < 0.01. **(E)** Mtb bacterial burden in the lung of Mtb-infected mice treated with PBS liposomes or clodronate liposomes i.v. (depletion of IMs). P-value was calculated using Student’s *t* test. *, P < 0.05. Error bars indicate standard deviation.

We hypothesized that if the different host macrophage lineages represent intracellular environments that are permissive (AMs) or restrictive (IMs) for Mtb survival and replication, then selective depletion of the two lineages should directly impact bacterial burden. We instilled clodronate-loaded liposomes intranasally to specifically deplete AM without impacting the number of IM and neutrophils (Fig. 5, A and B). Strikingly, the bacterial burden was reduced by over 80% in mice where AM had been depleted 2 wk after infection (Fig. 5 C). In a parallel experiment, we delivered clodronate-loaded liposomes intravenously to effectively deplete IM with minimal effect on the AM number and no impact on neutrophils (Fig. 5 D). In these IM-depleted animals, we observed the opposite phenotype, with an almost 10-fold increase in bacterial burden 2 wk after infection (Fig. 5 E). These results provide an independent dataset that supports our contention that AM and IM populations represent host cell populations that are differentially permissive to bacterial survival and growth.

### Alveolar and interstitial macrophages respond differentially to Mtb infection

**Figure 6.**
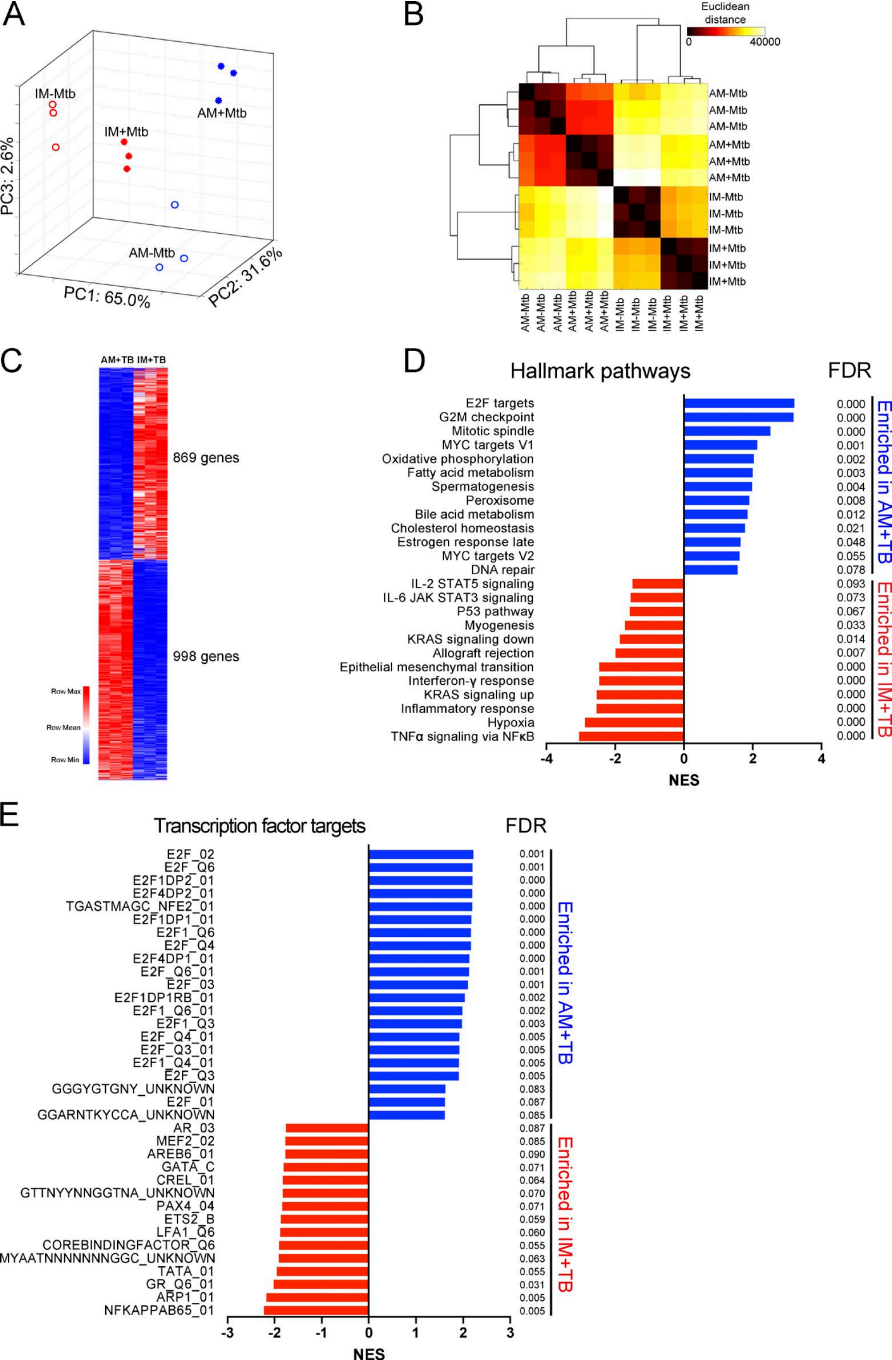
**Transcriptional profiles of AMs and IMs during Mtb infection.** RNA-sequencing analysis revealed distinct transcriptional profiles of AMs and IMs sorted from mice infected with mCherry-Mtb for 2 wk. AM+TB and IM+TB, cells with Mtb. AM-TB and IM-TB, cells without Mtb. **(A)** Principal-component (PC) analysis comparing AM-TB, AM+TB, IM-TB, and IM+TB. **(B)** Pairwise Euclidean distance with respect to the transcriptional profiles from each sample. **(C)** Heat map of differentially expressed genes between AM+TB and IM+TB. **(D)** GSEA of hallmark gene sets (H.all) from the Molecular Signatures Database of the Broad Institute, showing the most significantly enriched gene sets in AM+TB (blue) and IM+TB (red), their normalized enrichment scores (NESs), and false discovery rates (FDRs). **(E)** GSEA of transcription factor target gene sets (C3.TFT) from the Molecular Signatures Database of the Broad Institute, showing the most significantly enriched transcription factor target gene sets in AM+TB (blue) and IM+TB (red), their NESs, and FDRs. Data are from one experiment with three biological replicates per group obtained from four independent experiments.

To understand the physiological basis of the differential permissiveness of AMs and IMs for growth of Mtb, we performed global transcriptional profiling by RNA-sequencing of Mtb-infected and uninfected AMs and IMs isolated and flow-sorted 2 wk after infection with mCherry-expressing Mtb. RNA-sequencing data revealed that AMs and IMs with Mtb (AM+TB vs. IM+TB) had markedly distinct gene expression profiles, as illustrated by principal-component analysis (Fig. 6 A) and Euclidean distance calculation (Fig. 6 B). The uniqueness of the transcriptional profiles indicates that the two macrophage lineages respond differentially to Mtb infection. We identified 869 genes that were up-regulated in IM+TB more than twofold, whereas 998 genes were down-regulated in these cells compared with AM+TB (Fig. 6 C and Table S1). Macrophage properties are frequently aligned with in vitro cytokine-induced M1 and M2 phenotypes through the expression of specific polarization markers. As reported previously in other in vivo studies (Gibbings et al., 2017; Misharin et al., 2017), we found that these polarization markers did not segregate cleanly with the AM and IM populations (Fig. S3). We then performed GSEA and found the significant enrichment of several gene sets in AM+TB, such as targets of the family of transcription factors E2F, oxidative phosphorylation, and fatty acid metabolism in AM+TB. In contrast, gene sets involve in the inflammatory NF-κB response and hypoxia, a hallmark of glycolysis, were enriched in IM+TB (Fig. 6 D). Analysis of transcription factor binding sites also confirmed that genes containing E2F binding sites were highly enriched in AM+TB, whereas NF-κB binding sites were enriched in IM+TB (Fig. 6 E). E2F target genes have been shown to be involved in regulation of cell cycle, glycolysis and fatty acid metabolism, and mitochondrial function (Bracken et al., 2004; Benevolenskaya and Frolov, 2015). Therefore, perturbation of E2F expression has the capacity to increase Mtb’s access to host fatty acids and cholesterol, the preferred nutrients for intracellular bacilli (Lee et al., 2013; VanderVen et al., 2015). The data indicate that IMs mount a highly proinflammatory response to Mtb infection and that Mtb infection triggers markedly different physiological responses in the two macrophage lineages.

### AMs and IMs exhibit distinct metabolic states

The GSEA analysis above suggested that AMs and IMs infected with Mtb are in different metabolic states. Of note, Mtb infection induces glycolysis in mouse lungs and human macrophages, which has been linked to the control of Mtb growth (Shi et al., 2015; Gleeson et al., 2016). In addition, recent data indicate that the differential polarization of macrophages along the M1/M2 pathways also induce distinctive metabolic states (Van den Bossche et al., 2017).

**Figure 7.**
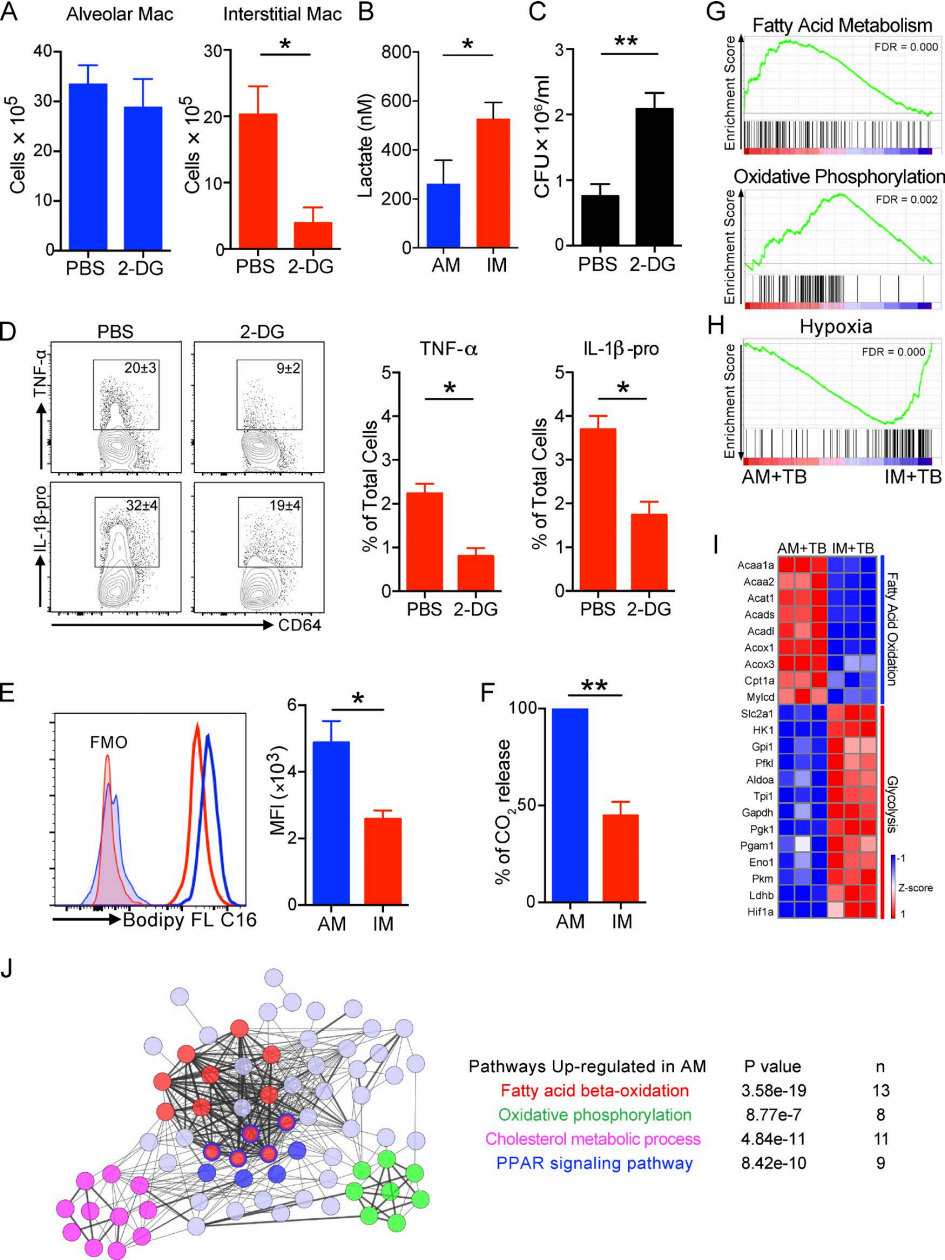
**Metabolic states of lung macrophage subsets in Mtb infection.** IMs are in a glycolytically active state, whereas AMs exhibit increased fatty acid uptake and β-oxidation of long chain fatty acids. Depletion of IMs through treatment with 2-DG leads to an increased bacterial burden, measured 2 wk after infection. **(A)** Numbers of AMs and IMs in mice treated with PBS or 2-DG. *n* = 4. The experiment was repeated two times. **(B)** Secreted lactate level in AMs and IMs after ex vivo culture for 36 h. The experiment was repeated two times. *n* = 2–3. Data were pooled from two experiments. **(C)** Mtb bacterial burden in the lung of Mtb-infected mice treated with PBS or 2-DG. *n* = 4. The experiment was repeated two times. P-values in A–C were calculated using Student’s *t* test. *, P < 0.05; **, P < 0.01. **(D)** Intracellular cytokine expression from IMs in Mtb-infected mice. Lung cells were ex vivo treated with PBS or 2-DG. Mean ± SD were shown in the flow plots to indicate the percentage of TNF-α and IL-1β producing IMs. Mean ± SD are shown in the column bar graph to indicate the percentage of TNF-α and IL-1β producing IMs in the total lung cells. *n* = 4. The experiment was repeated two times. P-value was calculated using Student’s *t* test. *, P < 0.05. **(E)** Histograms and MFI of Bodipy FL C16 in AM and IM in Mtb-infected mice. *n* = 3. The experiment was repeated two times. FMO, fluorescence minus one. P-value was calculated using Student’s *t* test. *, P < 0.05. **(F)** CO_2_ release from AMs and IMs sorted from Mtb-infected mice. *n* = 3. The experiment was repeated two times. P-value was calculated using Student’s *t* test. **, P < 0.01. **(G and H)** GSEA enrichment plots between AM+TB and IM+TB with false discovery rates (FDRs). **(I)** Heat map showing relative expression of genes in the fatty acid oxidation and glycolysis pathways in AM+TB and IM+TB. Data from RNA-sequencing analysis of three biological replicates in each group are shown. **(J)** Interactive network analysis of significantly up-regulated metabolic genes in AM+TB. Differentially expressed genes (fold change ≥ 1.5) in AM+TB versus IM+TB were analyzed to identify metabolic genes defined by the RGD gene database (http://rgd.mcw.edu/rgdweb/search/genes.html). The gene set was used to develop an interactive network of genes using STRING and further annotated and modified using Cytoscape. Thicker edges represent confidence >0.900. Pathways are color-coded. Each circle represents an individual gene. Genes with more than one color are components of more than one pathway. Error bars indicate standard deviation.

To functionally determine the metabolic status of the IM and AM phagocyte lineages during infection, we decided to probe the glycolytic activity of lung macrophages in Mtb infection in vivo, treating infected mice with 2-deoxy-d-glucose (2-DG) to block glycolysis. Treatment of mice with this nonhydrolyzable glucose analogue caused a significant reduction in IM numbers without impacting the number of AMs (Fig. 7 A), suggesting that IMs commit to glycolysis to sustain their survival, or enable recruitment to the lung, during infection in vivo. Furthermore, higher levels of lactate production were observed in ex vivo–cultured IMs than in AMs sorted from infected mice, which confirms the elevated glycolytic activity of IMs relative to AMs (Fig. 7 B). Importantly, the blockade of glycolysis also led to increased bacterial burden in the lung (Fig. 7 C), consistent with studies that indicate a requirement of this pathway in controlling Mtb growth (Shi et al., 2015; Gleeson et al., 2016). This result also provides independent validation of the impact of the reduction of IMs through clodronate-liposome depletion of circulating monocytes (Fig. 5, D and E). In addition, ex vivo secretion of TNF-α and IL-1β in response to Mtb infection was diminished in the presence of 2-DG in IMs (Fig. 7 D).

In contrast, Mtb is known to modulate host lipid metabolism to favor its survival in macrophages (Russell et al., 2009; Kim et al., 2010), where it is reliant on fatty acids and cholesterol as its primary carbon sources (Lee et al., 2013; VanderVen et al., 2015). We investigated fatty acid uptake in lung macrophages by injecting Mtb infected mice with fluorescently labeled long-chain fatty acid palmitate (Bodipy FL C_16_). Uptake of Bodipy FL C_16_ was higher in AMs than IMs (Fig. 7 E). To substantiate these findings further, we compared fatty acid oxidation in two lung macrophage subsets sorted from Mtb-infected mouse lungs by measuring CO_2_ release from cells cultured with media containing radiolabeled oleic acid. We found that AMs released significantly more radiolabeled CO_2_ than IMs under identical conditions (Fig. 7 F).

We further compared gene expression profiles between Mtb-infected AMs and IMs. GSEA revealed that genes involved in fatty acid metabolism and oxidative phosphorylation were the two positively enriched gene sets in Mtb-infected AMs, whereas a hypoxia signature was augmented in Mtb-infected IMs (and negatively enriched in AMs), supporting a shift toward glycolytic metabolism (Fig. 7, G and H). Mtb-infected AMs also revealed up-regulation of genes encoding proximal components of the fatty acid oxidation pathway. In contrast, genes regulating glycolysis were highly induced in IMs during Mtb infection (Fig. 7 I).

Lastly, we identified all genes related to metabolic pathways as defined by the RGD database (http://rgd.mcw.edu/rgdweb/search/genes.html) that had fold changes of expression >1.5 in Mtb-infected AMs over IMs. This gene set was then analyzed via Kyoto Encyclopedia of Genes and Genomes (KEGG) pathway enrichment, Gene Ontology (GO) analysis, and STRING, a database of known and predicted protein interactions. This analysis again showed fatty acid β-oxidation as the major metabolic network up-regulated in Mtb infection in AMs. Consistently, oxidative phosphorylation was also connected to this network (Fig. 7 J). These data reciprocate our experimental findings that AMs actively use fatty acids and engage in fatty acid metabolism. Interestingly, STRING also revealed cholesterol metabolism and PPAR signaling signatures in the network (Fig. 7 J).

These data demonstrate that in response to Mtb infection, the IM and AM populations in the murine lung tissue assume very different metabolic states. IMs are more glycolytically-active, behave like inflammatory macrophages, and exert control over Mtb growth and survival. In contrast, AMs are more active in fatty acid oxidation, acquire higher amounts of fatty acid, and present a preferred site for bacterial growth and survival.

### Glycolysis and fatty acid oxidation pathways in macrophages differentially control the growth of Mtb

**Figure 8.**
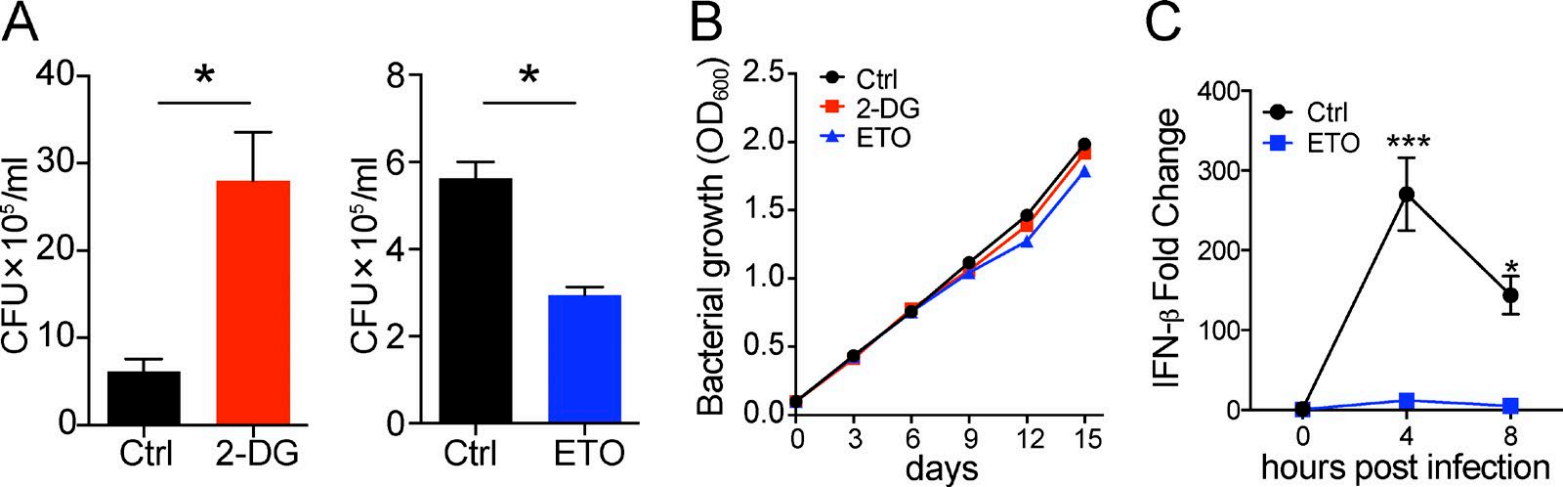
**Different metabolic pathways in macrophages regulate Mtb growth differentially in vitro.** Inhibition of glycolysis by 2-DG promotes Mtb growth in BMDMs, whereas inhibition of fatty acid oxidation by ETO suppresses Mtb growth. **(A)** CFU per milliliter 5 d after infection in BMDM treated with 0.1 mM 2-DG or 200 µM ETO or left untreated (Ctrl). The experiment was repeated three times. *n* = 3. P-values were calculated using Student’s *t* test. *, P < 0.05. Error bars indicate SEM. **(B)** Bacterial growth curve in 7H9/oleic acid/albumin/dextrose/catalase media with or without 0.5 mM 2-DG and 200 µM ETO. **(C)** Relative expression of IFN-β in Mtb Erdman–infected BMDMs in the presence of 200 µM ETO. The experiment was repeated two times. *n* = 3. P-values were calculated using Student’s *t* test. *, P < 0.05; ***, P < 0.001. Error bars indicate standard deviation.

Lastly, to establish a functional linkage between host macrophage metabolism and bacterial growth, we examined the impact of the metabolic inhibitors 2-DG (glycolysis) and etomoxir (ETO; fatty acid oxidation) on the growth of Mtb in murine bone marrow–derived macrophages (BMDMs). The inhibition of glycolysis with 2-DG enhanced bacterial growth, whereas ETO treatment reduced bacterial growth, as measured by CFU (Fig. 8 A). Neither 2-DG nor ETO had any effect on the growth of Mtb in broth culture (Fig. 8 B). These results indicate that the glycolytic pathway is required for limiting Mtb growth in macrophages, whereas fatty acid oxidation enhances the replication of intracellular bacilli. Recent studies have suggested that fatty acid metabolism is required for optimal type I interferon response (Wu et al., 2016), a pathway that is well known to promote Mtb pathogenesis (McNab et al., 2015). We found that Mtb induced the transcription of IFN-β in BMDMs, which peaked at 4 h after infection (Fig. 8 C). Surprisingly, production of IFN-β in Mtb-infected macrophages was nearly completely abolished in the presence of ETO (Fig. 8 C). Thus, fatty acid metabolism in macrophages is likely to be involved in regulating type 1 interferon response, which in turn further promotes the intracellular growth of Mtb.

## Discussion

Our ability to deal with tuberculosis as a threat to global health is compromised by the absence of an effective vaccine. The pathway to the development of such a vaccine is problematic, because we lack any reliable immune correlates of protective immunity (Bhatt et al., 2015; Goletti et al., 2016), and we cannot actually define the key components of a protective immune response capable of preventing disease progression in immune-competent individuals (Cadena et al., 2016).

We assume that disease progression is the consequence of loss of control, albeit at the localized level of the granuloma, and that this occurs in the absence of any systemic change in immune status, but this remains an unsubstantiated assumption. We believe that bacterial fitness reporters used in this current study represent a completely new approach to interrogating the dynamics of infection within experimental models. All immune mechanisms are filtered through the host phagocytes, and bacterial fitness reporters have the capacity to detect and respond to such pressures in a way that is immunologically agnostic, requiring no assumptions regarding mechanism, cytokine, or host cell phenotype.

We used an acute challenge model in mice to generate infected phagocytes that could be characterized with respect to their ability to control or promote bacterial growth, as well as their origin, developmental lineage, and metabolic status. This model is not intended to mirror disease progression but rather to generate sufficient infected macrophage populations for detailed experimental characterization of the link between macrophage physiology and bacterial fitness. 2 wk after infection, before the establishment of an adaptive immune response, nearly 40% of Mtb bacteria are distributed in lung AMs and IMs. The reporter Mtb strains indicate that bacilli in AMs experience less stress and maintain higher replication activity than those present in IMs. The functional significance of these data are demonstrated by the selective depletion of AMs, which negatively impacted bacterial burden, versus the depletion of circulating monocytes and recruited IMs, which increased the bacterial load in the infected lung. This change in bacterial burden is independent of an acquired immune response and is altered solely by modulating the size of the restrictive (IM) and permissive (AM) host phagocyte populations. These results are consistent with a previous study that reported that depletion of lung macrophages with different activation statuses has opposite effects on bacterial burdens in the lung (Leemans et al., 2005). Our current study adds further layers of information to this outcome through the use of bacterial fitness reporters to define the different macrophage lineages with respect to host cell physiology and bacterial permissiveness. Importantly, the monocytosis we observe here has also been reported in other studies using low-dose aerosol infection in mice and in active human tuberculosis (Antonelli et al., 2010; Wang et al., 2015; La Manna et al., 2017).

AMs and IMs represent two major populations of lung macrophages. Unlike AMs, functions of IMs and their roles in Mtb infection are much less clear (Jakubzick et al., 2017). Recent work has shown that IMs are capable of secreting IL-10 in response to bacterial CpG DNA stimulation and limiting allergic inflammation, suggesting a regulatory function of IMs (Sabatel et al., 2017). Our results showing that IMs actively produce IL-1β and TNF-α and are also iNOS positive in Mtb infection support the inflammatory properties of IMs. These data indicate that IMs are extremely plastic under different conditions. The distinct phenotypes of IMs could also be caused by heterogeneity within the IM population. Three unique IM subsets in the murine lung at steady state were identified recently; they differentially express CD206, MHCII, and CCR2 and exhibit unique transcriptional profiles (Gibbings et al., 2017). The contribution of IM subsets to promoting or controlling Mtb growth as well as disease progression merits further dissection.

**Figure 9.**
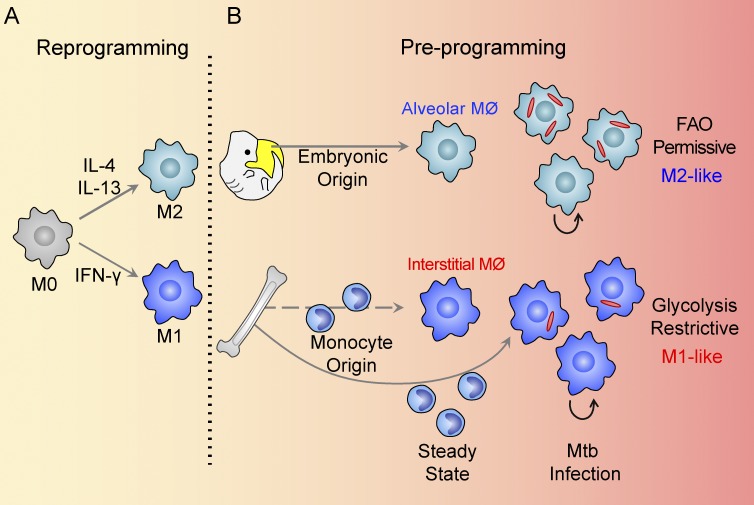
**Models of macrophage reprogramming and preprogramming. (A and B)** Schematic representation illustrating how macrophage function in the reprogramming model (A) is determined by immune signaling within the tissue niche. In the proposed preprogramming model (B), the function of coexisting macrophage lineages in the lung in Mtb infection is determined, in large part, by the origin of the macrophage. Published studies indicate that this metabolic bias between macrophage lineages exists before Mtb infection (Gibbings et al., 2017). FAO, fatty acid oxidation.

The impact of origin in determining macrophage functionality is a rapidly evolving area of research. The established M1/M2 paradigm of macrophage polarization emphasizes the importance of immune signals on macrophage activation. This model suggests that tissue environment rather than macrophage ontogeny is the main driver of macrophage functions. Several studies revealed that monocyte-derived macrophages are capable of adapting to a new tissue environment and converting to tissue resident macrophages (Scott et al., 2016; Gundra et al., 2017). More recently, a few studies have challenged this traditional view and provided evidence that macrophage origin plays a dominant role in the determination of functionality (Rückerl et al., 2017; Zhu et al., 2017). Our results are more consistent with those studies that demonstrate that lung macrophage populations from different origins mount divergent responses in Mtb infection, which are capable of either promoting or restricting bacterial growth. The data support a model of macrophage preprogramming, linked to its ontogeny, that exerts a dominant influence over the subsequent immune-mediated reprogramming during Mtb infection (Fig. 9). Furthermore, pathway analysis of transcriptional profiles of AMs and IMs from uninfected mice reveals up-regulated fatty acid metabolism in AMs, indicating the presence of distinct metabolic activities between AMs and IMs under normal homoeostasis (Gibbings et al., 2017). These data support the suggestion that there is a preexisting metabolic bias in these two macrophage populations. Understanding the impact of host cell heterogeneity and their relative responsiveness to immune-mediated control will be critical in avoiding the repetition of our past failures in vaccine development (Huang and Russell, 2017).

Our recently developed fluorescent Mtb reporter strains enabled us to quantify the relative fitness and replication status of Mtb in different phagocyte populations directly, which in turn revealed the permissiveness of the host phagocytes without perturbing phagocyte populations or immune response. The replication clock plasmid Mtb strain provided an independent validation of the data obtained from the Mtb reporter strains. The clock plasmid data enabled estimation of the rates of bacterial replication and death, which were calculated through the mathematical model developed by Gill et al. (2009). However, this analysis does make certain assumptions in the absence of information regarding the identity of the host cell infected before the host cell type is identified at the time of isolation. Nonetheless the strong consensus between the two disparate approaches argues that AMs represent a preferred site for bacterial replication in comparison with IMs.

Clodronate-loaded liposomes represent a powerful tool for the macrophage biology field to dissect functions of macrophages in various tissues by different administration routes (van Rooijen and Hendrikx, 2010). In the current study, we were able to deplete AMs by intranasal instillation and deplete blood monocytes, the precursors of IMs, by intravenous injection of clodronate liposomes. However, clodronate-mediated depletion of blood monocytes is not specific for IM depletion. Although we believe that much of the observed phenotype is a direct consequence of the depletion of IM precursors, it is possible that other immunologically active cells, such as the small number of CD11b^+^ dendritic cells, may modulate pathology even if they represent an insignificant proportion of host cells. Only 3% of the Mtb population were found in CD11b^+^ dendritic cells compared with the 80% of bacilli localized in neutrophils, AMs, and IMs (unpublished data).

Mtb infection induces glycolysis in mouse lungs and in human macrophages (Shi et al., 2015; Gleeson et al., 2016), and it is well established that in vitro M1 macrophages are dependent on glycolysis, whereas M2 macrophages are biased toward lipid uptake and fatty acid oxidation (O’Neill and Pearce, 2016; Van den Bossche et al., 2017). Our data provide more detailed in vivo insights, showing that IMs are engaged in glycolysis to produce inflammatory cytokines and are more adept at limiting bacterial growth. In contrast, AMs are more permissive, and are capable of producing type 1 interferons (Goritzka et al., 2015). Because Mtb bacteria are known to access and use host fatty acids and cholesterol (Lee et al., 2013; Podinovskaia et al., 2013; VanderVen et al., 2015), the metabolic status of the AM likely presents a nutritionally privileged niche. Recently, Marino et al. (2015) generated a predictive model of TB granuloma progression that was dependent on the relative abundance of M1 versus M2 macrophages at the site of infection. Much of the data from the model came from an earlier study that documented the differential expression and localization of nitric oxide synthases (eNOS and iNOS) versus arginases (Arg1 and Arg2), suggesting metabolically distinct microenvironments within the infected tissue (Mattila et al., 2013), which is supported by recent transcriptional profiling data (Marakalala et al., 2016). These data indicate that the distinct metabolic programing that we observed in the IM and AM populations in our murine model can be observed at the tissue level in nonhuman primates and human TB granulomas in vivo.

Consistent with previous acute challenge models (Srivastava et al., 2014), we also observed extensive neutrophil infiltration into sites of Mtb infection. The data on the function of neutrophils in Mtb infection are conflicting (Lowe et al., 2012; Dallenga and Schaible, 2016), and it is unclear whether neutrophils support or restrict bacterial replication. Our data obtained from the Mtb SSB-GFP reporter and clock plasmid strains indicate that Mtb are capable of actively replicating in neutrophils in vivo despite the high stress level revealed by the *hspX*::GFP reporter strain. This is in agreement with in vitro infection, although the infected neutrophils died shortly after infection (Corleis et al., 2012). A recent study reported that nitric oxide mediates the recruitment of neutrophils in Mtb infection, and further showed reduced bacterial loads in both NOS2^−/−^ and C3HeB/FeJ mice when neutrophils were specifically depleted (Mishra et al., 2017). These data are consistent with earlier studies indicating that tissue perturbations that lead to enhanced neutrophil recruitment also lead to increased bacterial burden and increased pathology (Kimmey et al., 2015; Mattila et al., 2015). Altogether, these data suggest that neutrophils are permissive host cells, in agreement with the observation that neutrophils containing abundant Mtb are readily isolated from active human tuberculosis cases (Eum et al., 2010). Undoubtedly the role(s) of the neutrophil in tuberculosis infection merits greater attention; however, in the current study the modulation of bacterial burden through selective depletion of IM and AM is achieved without detectable impact on the neutrophil population. Although the influence that neutrophils may exert over the immune milieu that programs the IM and AM populations undoubtedly requires further examination.

Macrophages have been shown to be capable of proliferating in situ under various inflammatory conditions (Jenkins et al., 2011; Robbins et al., 2013; Blériot et al., 2015). As a hallmark of Mtb infection, the granuloma is composed of large numbers of macrophages. We show that Mtb infection induces proliferation of both AM and IM. The expression of the replication marker Ki67 is mutually exclusive to both Mtb infection and to iNOS expression in both IM and AM, suggesting Mtb infection, at the cellular level, leads to an arrested cell cycle, although we cannot formally exclude the alternative explanation that Mtb preferentially infects nondividing cells. The contribution of the replication of bystander uninfected lung macrophages to the total phagocyte population and to the evolution of the infection site during the course of disease remains to be assessed.

The goal of this study was to provide an alternative perspective for the biological processes that promote active tuberculosis. In general, active disease is considered to be the loss of immune-mediated control, which provides a rationale for vaccine development that emphasizes the induction of immune responses linked to control (Tanner et al., 2016). But TB disease progression could equally be caused by gain of permissiveness, namely the preferential expansion of host cells disposed to support bacterial growth and replication. This argument is not a tautological one if permissiveness is an immunological property that is independent of control. The data from this study demonstrate the differential permissiveness of the two major macrophage lineages present in the mouse lung during acute infection and provide a potential mechanism whereby expansion of permissive AMs could promote bacterial growth, even in the face of a controlling immune response.

Translating these findings into avenues to improve therapy could include host-directed therapeutic approaches to minimize expansion of permissive macrophages during infection or the utilization of drug regimens that incorporate newly identified inhibitors of Mtb cholesterol metabolism (VanderVen et al., 2015) to target these bacilli specifically. Although this picture remains daunting, we believe that the new insights into immune-modulated metabolism of the infected host cell subsets, together with the ability to preferentially target bacterial subpopulations within the site of infection, provide us with the means to meet these challenges.

## Materials and methods

### Mice

C57BL/6 WT, Cyclin B1-GFP, CCL2^−/−^, Rag2^−/−^, and CD45.1 mice were purchased from The Jackson Laboratory. Mice were used at 6–8 wk old. All mice were maintained in a specific pathogen–free biosafety level-3 facility at Cornell University. Sex- and age-matched controls were used in all experiments. Animal care was in accordance with the guidelines of the Association for Assessment and Accreditation of Laboratory Animal Care. All animal procedures were approved by the Institutional Animal Care and Use Committee of Cornell University.

### Mtb strains

The Erdman strain of Mtb was used in the study. Erdman (*smyc’*::mCherry) and two fluorescent reporter Mtb strains, Erdman (SSB-GFP, *smyc’*::mCherry) and Erdman (*hspX’*::GFP, *smyc’*::mCherry), have been previously described (Tan et al., 2013; Sukumar et al., 2014). Mtb Erdman WT was transformed with the plasmid pBP10 as described previously (Gill et al., 2009). The bacteria were grown to log phase in Middlebrook 7H9 broth supplemented with 10% oleic acid/albumin/dextrose/catalase (OADC), 0.2% glycerol, 0.05% Tween 80, and 50 µg/ml hygromycin B (previously published mCherry and two reporter Mtb strains) or 30 µg/ml kanamycin (Mtb strain-pBP10), aliquoted in 10% glycerol, and stored at −80°C until use.

### Mice infections with Mtb

Mice were intranasally inoculated with ∼1,000 CFUs of different Erdman strains in 25 µl PBS containing 0.05% Tween 80. The inoculum dosage was confirmed by plating different dilutions on 7H10 plates supplemented with 50 µg/ml hygromycin B. Bacterial loads were determined by homogenizing the left lung lobe together with the accessory lobe of the right lung in PBS containing 0.05% Tween 80 and plating on 7H10 agar. Plates were incubated at 37°C and colonies enumerated 3 wk after.

### Lung cell isolation

Lung cells were isolated as described previously (Liu et al., 2016). Briefly, lungs were removed, minced, and digested in 5% FBS/PBS solution containing 250 U/ml collagenase IV (Worthington) and 20 U/ml DNase (Roche) for 60 min at 37°C. The digested lung material was then pass through a 70-µm cell strainer. Red blood cells were lysed with ACK lysis buffer (Lonza). Lung cells isolated for the RNA-sequencing study were generated using a gentle MACS dissociator (Miltenyi Biotec).

### Flow cytometry and sorting of lung phagocytes

Lung cell suspensions were incubated with Fc block (eBioscience) for 15 min. Cells were then counted and incubated for 30 min in the dark with fluorophore-conjugated antibodies or isotype control antibodies. Fluorochrome-conjugated mAbs specific to mouse CD11b (M1/70; BD Biosciences), CD11c (N418), CD115 (AFS98), CD45.1 (A20), CD64 (X54-5/7.1; BioLegend), CX_3_CR1 (SA011F11; BioLegend), F4/80 (BM8), FcεRIα (MAR-1; BioLegend), IL-1β (NJTEN3), iNOS (CXNFT), Ki67 (SolA15), Ly6C (AL-21; BD Biosciences), Ly6G (1A8; BioLegend), MerTK (DS5MMER), MHCII (M5/114.15.2), SiglecF (E50-2440; BD Biosciences), and TNFα (MP6-XT22; BD Biosciences), and fixable viability dye were purchased from eBioscience unless otherwise indicated. The Foxp3/Transcription Factor Staining buffer set (eBioscience) was used for Ki67 staining. To measure Bodipy FL C_16_ uptake in vivo, mice were injected i.p. with 50 µg Bodipy FL C_16_ per mouse diluted in DMSO. Lung cells were isolated 60 min after injection. Cells were analyzed with an LSRII flow cytometer (BD). For reporter Mtb strain quantification, fixed lung cells were sorted with a FACSAria machine (BD) in the Flow Cytometry Core at Cornell University. Data were analyzed with FlowJo software (Tree Star).

### Adoptive monocyte transfer

Monocytes were purified from bone marrow of uninfected CD45.1 mice using a monocyte isolation kit (BM; Miltenyi Biotec). The mean purity of monocytes from this procedure was >93% (Ly6C^+^CD11b^+^). 5 × 10^5^ monocytes were resuspended in 200 µl sterile PBS and injected intravenously into recipient mice 10 or 24 d after infection.

### Depletion of blood monocytes and alveolar macrophages

Clodronate-loaded and PBS-loaded liposomes were purchased from clodronateliposomes.com. For depletion of blood monocytes, mice were injected intravenously with 200 µl clodronate-loaded or PBS-loaded liposomes every 2 d. For depletion of alveolar macrophages, mice were treated intranasally with 100 µl clodronate-loaded or PBS-loaded liposomes every day for the first week and every 2 d for the second week.

### Labeling of blood Ly6C^hi^ monocytes

Specific labeling of blood Ly6C^hi^ monocytes was performed as described previously (Tacke et al., 2006). Briefly, 250 µl clodronate-loaded liposome were injected intravenously to deplete peripheral blood monocytes, followed by intravenous injection of 250 µl FITC-conjugated plain microspheres (1.0 µm, diluted 1:4 in sterile PBS; Polysciences) 18 h later. Lungs were processed for flow cytometry analyses 3 d after bead injection (14 d after infection).

### Quantification of Mtb reporter expression

Sorted lung phagocytes were stained with DAPI and Alexa Fluor 647–conjugated phalloidin (Invitrogen) and attached on poly-l-lysine–coated slides. Reporter Mtb expression quantification was performed as previously described (Sukumar et al., 2014). Briefly, cells were imaged using a Leica SP5 confocal microscope. Z-stacks were reconstructed into 3D using Volocity software (PerkinElmer) and Mtb GFP expression quantified by simultaneously measuring the voxel volume of each bacterium, via the mCherry channel, and the corresponding sum of the GFP signal for that bacterium. Quantification of the SSB-GFP reporter was accomplished by counting the number of bacteria with and without-SSB-GFP replication foci and calculating the total percentage of bacteria presenting one or more SSB-GFP foci for each cell type. At least 100 bacteria were quantified.

### Replication clock plasmid Mtb infection

Lung phagocytes were isolated from Erdman (Mtb strain-pBP10)–infected mice and sorted using a S3 cell sorter (Bio-Rad Laboratories). The purity of sorted cells was at least 95%. Sorted cells were pelleted and lysed in 0.01% SDS/H_2_O. Aliquots were plated on 7H10 agar plates ±30 µg/ml kanamycin. CFUs were enumerated to calculate the loss of kanamycin resistance during the course of infection. Rates of replication and death, generation time, and numbers of dead bacteria were quantified using the mathematical model as described previously (Gill et al., 2009). Specifically, in the supplemental materials of Gill et al. (2009), their mathematical modeling process was detailed and 13 equations listed. Rate of replication (r) was calculated using equation 10, rate of death (δ) was calculated using equation 11, and numbers of dead bacteria were calculated using equation 13. Generation time was calculated using the following equation as described in Table 1 of Gill et al. (2009): generation time = ln(2)/r. Mtb segregation constant (s = 0.18) determined in the original paper was used for all the calculations in the current study.

### RNA sequencing

RNA isolation and sequencing were done at the RNA Sequencing Core at Cornell University. Briefly, total RNA was extracted from FACS-purified lung macrophages using Trizol according to the commercial protocol, with the addition of a chloroform back-extraction step, addition of GlycoBlue (Thermo Fisher) as a carrier at the precipitation step, and a second 70% ethanol wash of the RNA pellet. All RNA samples (three biological replicates in each group) passed quality control analysis on a Fragment Analyzer (Advanced Analytical). Directional poly-A^+^ RNA-sequencing libraries were generated with the NEBNext Ultra II RNA library prep kit (New England BioLabs). Libraries were sequenced on a NextSeq500 (Illumina) for a mean of 41 million reads per sample. The Tophat program (version 2.1) was used to align the reads to the UCSC mm10 mouse reference genome, and the Cufflinks program (version 2.2) was used for the measurements of transcript abundance represented by fragments per kilobase of transcript per million mapped reads and statistical analysis of differential gene expression. RNA-sequencing data have been deposited under GEO accession no. GSE108844.

### RNA-sequencing data analysis

Principal-component analysis and Euclidean distance were calculated using MATLAB. To identify the enriched gene signatures, we used the GSEA tool available from the Broad Institute website. We used 1,000 gene-set permutations for testing of significance. We screened the collection of signatures under the categories H (hallmark gene sets), C2.CP (canonical pathways gene sets), C3.TFT (transcription factor targets), and C5 GO (GO gene sets). Interactive network was constructed as described previously (Wu et al., 2016). Briefly, differentially expressed genes (fold change ≥ 1.5) in AM+TB versus IM+TB were analyzed. Searching with the key words metabolism, glycolysis, fatty acid oxidation, fatty acid uptake, fatty acid synthesis, oxidative phosphorylation, lipid uptake, lipid synthesis, cholesterol synthesis, cholesterol metabolism, cholesterol homeostasis, and cholesterol efflux, differentially expressed genes in each metabolic pathway, as defined by the RGD gene database (http://rgd.mcw.edu/rgdweb/search/genes.html), were identified. Genes were then archived for network analysis. STING10 was used to constructed a network of interactions. The resulting network of genes was annotated and modified in Cytoscape. Pathway enrichment analysis based on KEGG and GO Biological Process was performed, and significantly enriched pathways based on low p-values and false discovery rates were used for further analysis.

### BMDM culture and in vitro assays

BMDMs were isolated from C57BL/6J mice and cultured in DMEM (Corning cellgro) supplemented with 10% FBS (Thermo Scientific), 20% L-cell conditioned media, 2 mM l-glutamine, 1 mM sodium pyruvate, and 1% penicillin/streptomycin (Corning cellgro) at 37°C for 6 d. BMDMs were infected with Mtb Erdman at multiplicity of infection 2 for 3 h, before extracellular bacteria were removed. Fresh media with or without 0.1 mM 2-DG or 200 µM ETO (Cayman) were added. For CFU determination, cells were lysed with 0.01% SDS in water and plated on 7H10 agar.

### Quantitative RT-PCR analysis

Total RNA was isolated from Mtb Erdman–infected BMDMs treated with or without 200 µM ETO at multiplicity of infection 10 at indicated time points using a RNeasy Mini kit (QIAGEN). Reverse transcription of 1 µg RNA was performed using a iScript cDNA synthesis kit (Bio-Rad). Quantitative RT-PCR was performed for IFN-β using the Applied Biosystems PRISM 7500 Sequence Detection System and its analysis software, SDS 2.3 and RQ Manager. HPRT was used as the housekeeping gene. The reactions were performed using the TaqMan Universal PCR Master Mix. All primers were purchased from Applied Biosystems.

### In vivo and ex vivo inhibitor treatment

To block glycolysis, mice were administered 5 mg 2-DG per mouse every other day starting upon infection for 14 d. For ex vivo treatment, isolated lung cells were cultured in the presence of 10 mM 2-DG and Brefeldin A (eBioscience) for at least 4 h. Surface and intracellular staining with antibodies described above were performed using eBioscience Intracellular Fixation and Permeabilization buffer set.

### Lactate measurement

AMs and IMs were sorted from Mtb-infected mice 2 wk after infection as described above. 10^5^ cells of each population were seeded in 96-well plates in DMEM with 10% FBS. Supernatants were deproteinized by filtering through a 10-kD molecular weight spin filter (BioVision). Lactate level was quantified using a lactate colorimetric/fluorometric assay kit (BioVision).

### Fatty acid oxidation assay

To determine fatty acid oxidation activity in macrophages, equal numbers of alveolar and interstitial macrophages from infected mouse lungs were sorted using a S3 cell sorter (Bio-Rad Laboratories) and cultured in RPMI1640 media containing 1.0 µCi [1-^14^C]-oleic acid (PerkinElmer). Each Petri dish was placed with an open vial of 500 µl of 1N NaOH in an air-tight jar and incubated at 37°C. The vial of NaOH was removed after 24 h and neutralized with an equal volume of 1N HCl. The amount of solubilized ^14^CO_2_ was quantified by scintillation counting (LS6500; Beckman Coulter).

### Statistical analysis

Statistical information, including *n*, mean, and statistical significance values, is indicated in the figure legends. Results were statistically analyzed using Student’s *t* test or one-way ANOVA test with multiple comparisons where appropriate using Prism 6.0 (GraphPad Software).

### Online supplemental materials

Fig. S1 shows flow cytometry gating strategy for mouse lung phagocytes 2 wk after Mtb infection. Fig. S2 (related to Fig. 3) shows percentages of Ki67^+^ and iNOS^+^ cells in AM and IM populations in WT and Rag2^−/−^ mice 2 wk after Mtb infection. Fig. S3 (related to Fig. 6) shows the heat map of relative expression of M1/M2 polarization markers, chemokines, and chemokine receptors in AM+TB and IM+TB. Table S1 (related to Fig. 6) contains list of differentially expressed genes in AM+TB and IM+TB and is included as an Excel file.

## Supplementary Material

Supplemental Materials (PDF)

Table S1 (Excel file)
