# Supplementary material for: Growth of Mycobacterium tuberculosis in vivo segregates with host macrophage metabolism and ontogeny
Source: J Exp Med. 2018 Apr 2;215(4):1135–52. doi: 10.1084/jem.20172020 (PMC5881470; doi:10.1084/jem.20172020)
Supplement: Supplemental Materials (PDF) [file JEM_20172020_sm.pdf]

SUPPLEMENTAL MATERIAL

Huang et al., https://doi.org/10.1084/jem.20172020

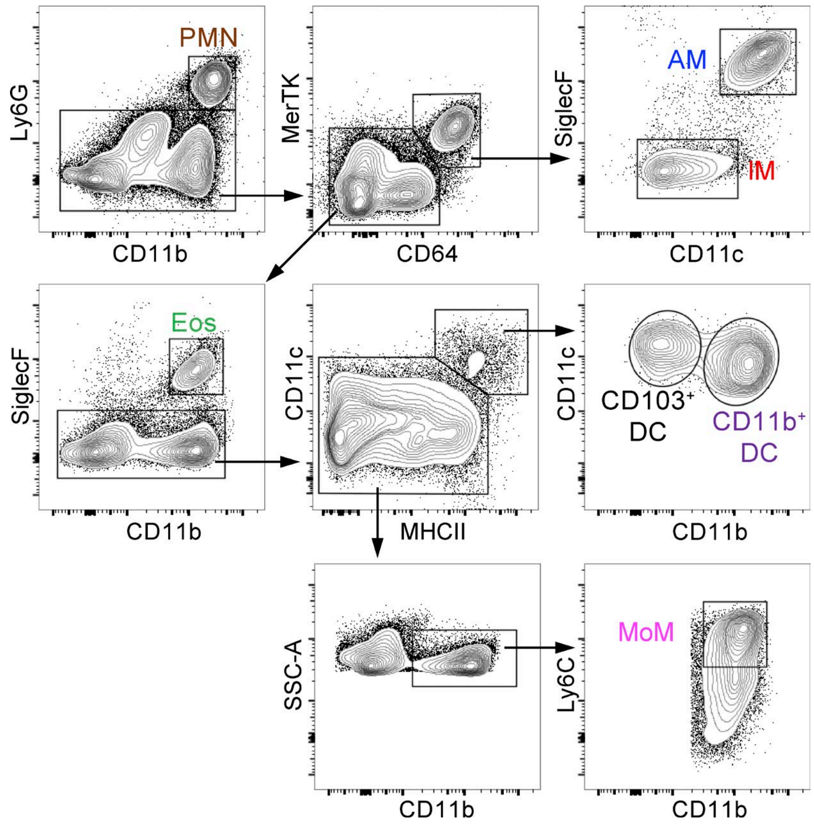

Figure S1. **Accumulation of interstitial macrophages in Mtb infection (related to Fig. 1).** Gating strategy for identification of lung phagocytes. DC, dendritic cell; Eos, eosinophil; MoM, monocyte; PMN, neutrophil.

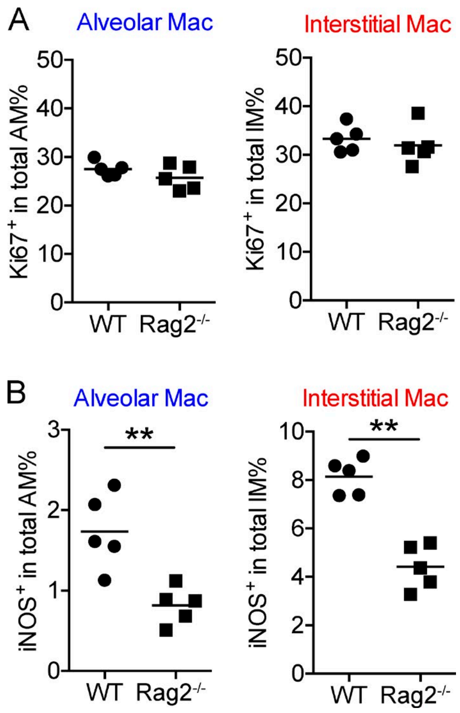

Figure S2. **Mtb induces local proliferation of lung macrophages (related to Fig. 3).** Flow cytometry analysis of lung macrophages. **(A and B)** Percentages of Ki67⁺ cells (A) and iNOS⁺ cells (B) out of AMs and IMs. WT and $Rag2^{-/-}$ mice were infected with 10^3 Mtb for 2 wk. *n* = 5. The experiment was repeated two times. P-values were calculated using Student's *t* test. **, P < 0.01. Horizontal bars indicate the mean.

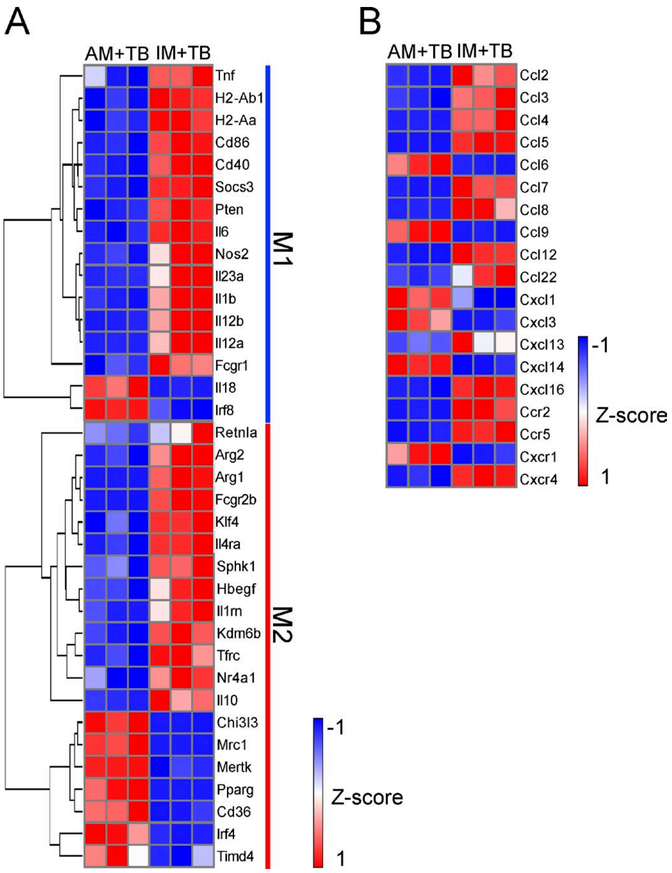

Figure S3. **Transcriptional profiles of AMs and IMs in Mtb infection (related to Fig. 6). (A and B)** Heat map showing relative expression of M1/M2 polarization markers (A) and chemokines and chemokine receptors (B) in AM+TB and IM+TB. Data from RNA-sequencing analysis of three biological replicates in each group are shown.

Table S1 (related to Fig. 6) contains list of differentially expressed genes in AM+TB and IM+TB and is included as an Excel file.
